# Liquid-Phase Synthesis and Regulatory Mechanisms of Nano-Nickel Powders for MLCC Inner Electrodes

**DOI:** 10.3390/nano16080491

**Published:** 2026-04-21

**Authors:** Zhenzong Quan, Jianwei Wang, Huijun He, Xingming Wang, Liqing Ban, Xiaoling Ma, Haijun Zhao

**Affiliations:** 1 General Research Institute for Nonferrous Metals, Beijing 100088, China; jushu4840@gmail.com (Z.Q.); wxm@grinm.com (X.W.); banliqing@gripm.com (L.B.); maxiaoling12303@126.com (X.M.); hjzhao2023@163.com (H.Z.); 2 GRINM Advanced Materials Co., Ltd., Beijing 100088, China; 3 GRINM NEXUSX Advanced Materials (Beijing) Co., Ltd., Beijing 101407, China

**Keywords:** nano-nickel powder, liquid-phase reduction, MLCCs, morphological control, HCP-Ni, functional additives

## Abstract

Driven by the demand for miniaturization, high capacitance, and enhanced reliability in high-performance multilayer ceramic capacitors (MLCCs), the continuous thinning of inner electrode layers imposes increasingly stringent requirements on the size, distribution, morphology, and dispersion of nano-nickel powders. We systematically investigate how functional additives regulate the nucleation, growth, and microstructural evolution of nano-nickel synthesized via hydrazine-driven liquid-phase reduction of nickel sulfate. The results demonstrate that the alkanolamine complexing agent (TAC) significantly refines the average particle size and morphology of the nano-nickel through coordination effects. Furthermore, inorganic sulfur salts (ISP), acting via surface adsorption to passivate growth sites and provide catalytic effects, enable a precise and continuous reduction in the average particle diameter from 330 nm down to 60 nm at a mere trace dosage of ~10^−7^ mol/L. Regarding dispersion optimization, highly dispersed face-centered cubic (FCC) nano-nickel was successfully prepared by introducing multidentate carboxylate (NNA). High-resolution transmission electron microscopy (HRTEM) was employed to unveil, for the first time, the crystallographic origin of the anomalous surface protrusions typically observed in conventional reaction systems. We confirmed that the family of $\left\{10\bar{1}0\right\}$ crystal planes within these regions, which exhibits interfacial angles of 58.7° and 58.3°, corresponds to a thermodynamically metastable hexagonal close-packed (HCP) nickel phase originating from atomic stacking faults induced by rapid growth kinetics. To address this microstructural defect, a thioether-based amino acid (TAA) was introduced. TAA effectively suppresses the anisotropic growth of the metastable HCP phase through the strong steric hindrance of its long side chains and its selective adsorption onto high-energy facets.

## 1. Introduction

As core fundamental components for energy storage and signal coupling, multilayer ceramic capacitors (MLCCs) are essential components in 5G communications, automotive electronics, the Internet of Things (IoT), and aerospace [1,2,3]. Driven by the trend toward miniaturization and high integration in electronic devices, the continuous thinning (<1 μm) and densification of MLCC inner electrode layers impose extremely stringent requirements on conductive powders [4,5]. The large-scale application of traditional noble metals (Pd/Ag) is severely restricted by their high costs [6]. In contrast, metallic nickel (Ni) has emerged as the mainstream material for next-generation base metal electrodes (BMEs) owing to its excellent electrical conductivity, superior electromigration resistance, and significant cost advantages [7,8,9]. To meet the requirements for low-temperature co-firing with BaTiO_3_-based dielectric layers in a reducing atmosphere [10,11,12], nanoscale nickel powders must simultaneously exhibit ultra-fine and controllable particle sizes, narrow size distributions, high sphericity, and excellent dispersibility. The quality of these powder precursors directly determines the dielectric properties and reliability of the final MLCC devices [13,14], The surface condition of the particles generally influences their reactivity during the co-firing process [15]. Particle size refinement is conducive to overcoming the electrode thickness limit to achieve high capacitance, retarding sintering shrinkage, and ensuring a better match with the dielectric layers. Nickel powders with excessively large particle sizes or non-uniform size distributions are prone to generating heterogeneous stresses during sintering, ultimately leading to delamination and cracking within the MLCCs [1]. Jo et al. [16]. indicated that severe particle agglomeration results in low electrode packing density and an increased risk of short circuits; reducing the agglomeration ratio of the nickel powders to below 4.8% can significantly enhance layer thickness precision and mitigate short circuit failures. Furthermore, Im et al. [13] demonstrated that uniform particle sizes and high sphericity can decelerate the sintering rate, thereby reducing capacitance noise by 25%.

Among primary synthesis techniques (e.g., CVD, plasma synthesis, microemulsion) for nano-nickel, liquid-phase reduction exhibits broad industrial prospects owing to its modest equipment requirements, tunable parameters, and scalability [17,18]. This approach typically employs strong reducing agents to reduce nickel precursors into elemental metal nanoparticles within a liquid system [19,20,21]. However, the liquid-phase reduction system involves highly complex homogeneous and heterogeneous nucleation, coupled with complicated interfacial growth kinetics. Driven by extremely high specific surface energies and intrinsic magnetic interactions, nickel nanoparticles readily agglomerate [22]. Moreover, anisotropic crystal growth frequently leads to irregular morphologies featuring urchin-like or spiky surface protrusions. Previous studies have predominantly focused on optimizing process parameters or introducing dispersants [23,24], but existing research is largely confined to the physical steric isolation provided by single-type dispersants (particularly polymers), with insufficient attention given to additives capable of modulating the reaction pathways at the fundamental chemical level. Furthermore, a systematic investigation into the synergistic regulatory mechanisms of such functional additives within the hydrazine-driven nickel sulfate reduction system remains conspicuously absent.

In this study, we propose a strategy to modulate the thermodynamics and kinetics of the liquid-phase reduction process via the introduction of multi-component functional additives, thereby achieving high-precision microstructural design of nano-nickel powders. Utilizing a base reaction system of nickel sulfate and hydrazine, we selected an alkanolamine complexing agent (TAC), an inorganic sulfur salt (ISP), a multidentate carboxylate (NNA), and a thioether-based amino acid (TAA) as targeted modifiers. By systematically investigating their impact on the morphology, particle size, and dispersibility of the synthesized nano-nickel, we aim to elucidate the underlying mechanisms by which these distinct classes of additives govern the nucleation, growth, and agglomeration processes. This work presents a controllable synthetic strategy for producing spherical, monodisperse nano-nickel powders tailored for high-performance MLCCs, and provides an experimental framework for understanding additive-mediated growth in liquid-phase reduction systems.

## 2. Experimental

### 2.1. Materials

Nickel sulfate hexahydrate (NiSO_4_·6H_2_O, 98.5%, Shandong Keyuan Biochemical Co., Ltd., Heze, China) was utilized as the primary nickel precursor. Other reagents included a multidentate carboxylate (NNA, 99% analytical grade, Shanghai Aladdin Biochemical Technology Co., Ltd., Shanghai, China), sodium hydroxide (NaOH, 95%, Shandong Keyuan Biochemical Co., Ltd., Heze, China), an analytical grade alkanolamine complexing agent (TAC, Shanghai Aladdin Biochemical Technology Co., Ltd., Shanghai, China), hydrazine hydrate (N_2_H_4_·H_2_O, 85%, Sinopharm Chemical Reagent Co., Ltd., Shanghai, China), an inorganic sulfur salt (ISP, 99%, Sinopharm Group Co., Ltd., Shanghai, China), and a thioether-based amino acid (TAA, 99%, Shanghai Aladdin Biochemical Technology Co., Ltd., Shanghai, China).

### 2.2. Synthesis Procedure

A predetermined amount of the nickel precursor ($\mathrm{Ni}{\mathrm{SO}}_{4}\cdot 6H_{2}O$) and the multidentate carboxylate (NNA) were placed into a three-neck flask, followed by the addition of 190 mL of deionized water. The resulting mixture was subsequently heated to 72 °C. Separately, a specific volume of hydrazine hydrate solution ($N_{2}H_{4}\cdot H_{2}O$) and the alkanolamine complexing agent (TAC) solution were pre-mixed in a beaker and diluted with 20 mL of deionized water. Once the primary nickel precursor solution reached 70 °C, this reducing mixture was poured into the flask, vigorously stirred, and heated for 10 min. During this stage, the macroscopic color of the solution was observed to shift depending on the applied molar ratio of hydrazine to nickel ions (N_2_H_4_/Ni^2+^). A precise quantity of the inorganic sulfur salt (ISP) was dissolved in 100 mL of deionized water under continuous stirring and stored in a sealed reagent bottle. A high-precision micropipette (5–20 μL) was utilized to transfer required trace amounts of this ISP solution into the reaction system. Concurrently, a specific mass of solid sodium hydroxide (NaOH) was dissolved in 50 mL of deionized water, heated to 60 °C, and then rapidly introduced into the main reaction mixture under continuous agitation. Upon completion of the reaction, the flask was immediately transferred to a cold-water bath to rapidly quench the mixture to room temperature. The resulting precipitates were dispersed using an ultrasonic cell disruptor and subsequently collected via centrifugation; this washing cycle was repeated 4 to 5 times until the supernatant became entirely transparent. The purified samples were finally dried in a vacuum oven at 60 °C for 24 h prior to being sealed for storage. For experimental groups involving dispersion via the thioether-based amino acid (TAA), a pre-configured TAA solution was introduced approximately 1 min and 30 s after the macroscopic observation of the solution turning black.

### 2.3. Characterization

The morphology and particle size of the samples were characterized using a field-emission scanning electron microscope (FESEM, JSM-7900F, JEOL Ltd., Tokyo, Japan). The particle size distribution and relative content were determined via a wet-dispersion method utilizing a Bettersize 2600 laser particle size analyzer(Bettersize Instruments Co., Ltd., Dandong, China). X-ray diffraction (XRD) patterns of the samples were recorded using a diffractometer (SmartLab, Rigaku Corporation, Tokyo, Japan) equipped with a Cu Kα radiation source (λ = 1.5418 Å). Fourier-transform infrared (FTIR) spectra were collected over a wavenumber range of 4000 to 400 cm^−1^ using an FTIR spectrometer (VERTEX 70, Bruker Optics Inc., Billerica, MA, USA). Detailed microstructural characterization was performed using a transmission electron microscope (TEM, JEM-2010, JEOL Ltd., Tokyo, Japan). The elemental valence states on the surface of the nano-nickel particles were characterized using an X-ray photoelectron spectrometer (XPS, ESCALAB Xi+, Thermo Fisher Scientific, Waltham, MA, USA).

## 3. Results and Discussion

### 3.1. Reaction Mechanism

In this reaction system, hydrazine (N_2_H_4_) serves as the primary reducing agent. Because the reduction potential of N_2_H_4_ is more negative in alkaline media than in acidic media, the syntheses were conducted under alkaline conditions. The corresponding half-reactions are expressed in Equations (1)–(3).

(1) $$N_{2}+4H_{2}O+4e^{-}\to N_{2}H_{4}+4{OH}^{-}\;E_{25}^{{}^{\circ}}\;=-1.16\;V\;E_{70}^{{}^{\circ}}\;=-1.21\;V$$

(2) $${Ni}^{2+}+2e^{-}\to Ni\;E_{25}^{{}^{\circ}}\;=-0.257\;V\;E_{70}^{{}^{\circ}}\;=-0.22\;V$$

(3) $${Ni}^{2+}+2{OH}^{-}\to Ni{\left(OH\right)}_{2}\;Ksp=6\times 10^{-16}$$

Upon mixing the reducing mixture with the nickel precursor solution, a complex precipitate, $\left[\mathrm{Ni}{\left(N_{2}H_{4}\right)}_{n}\right]{\mathrm{SO}}_{4}$, initially forms, as described by Equation (7). However, this complex is thermodynamically unstable and undergoes a ligand exchange reaction with OH^−^ in the solution (Equation (8)), thereby re-releasing N_2_H_4_ and yielding Ni(OH)_2_. Subsequently, the Ni(OH)_2_ is reduced to elemental nickel according to Equation (4).

(4) $$\mathrm{Ni}{\left(OH\right)}_{2}+2e^{-}\to Ni+2{OH}^{-}E_{25}^{{}^{\circ}}\;=-0.72\;V\;E_{70}^{{}^{\circ}}\;=-0.737\;V$$

(5) $$2H_{2}O+2e^{-}\to H_{2}+2{OH}^{-}E_{25}^{{}^{\circ}}\;=-0.828\;V\;E_{70}^{{}^{\circ}}\;=-0.835\;V$$

(6) $$N_{2}H_{4}+2H_{2}O+2e^{-}\to 2NH_{3}+2{OH}^{-}\;E_{25}^{{}^{\circ}}\;=+0.1\;V\;E_{70\;}^{{}^{\circ}}=+0.119\;V$$

(7) $$Ni{SO}_{4}+nN_{2}H_{4}\to \left[Ni{\left(N_{2}H_{4}\right)}_{n}\right]{SO}_{4}$$

(8) $$\left[Ni{\left(N_{2}H_{4}\right)}_{n}\right]{SO}_{4}+2{OH}^{-}\to Ni{\left(OH\right)}_{2}+nN_{2}H_{4}+{{SO}_{4}}^{-}$$

Combining all the relevant half-reactions yields the overall reaction equations, presented as Equations (9) and (10). Taking into account the inherent side reactions of hydrazine, an excess of hydrazine is typically required to ensure the complete reduction of nickel ions within the system.

(9) $${Ni}^{2+}+2{OH}^{-}\to Ni{\left(OH\right)}_{2}$$

(10) $$2Ni{\left(OH\right)}_{2}+N_{2}H_{4}\to 2Ni+N_{2}+4H_{2}O$$

**Figure 1:**
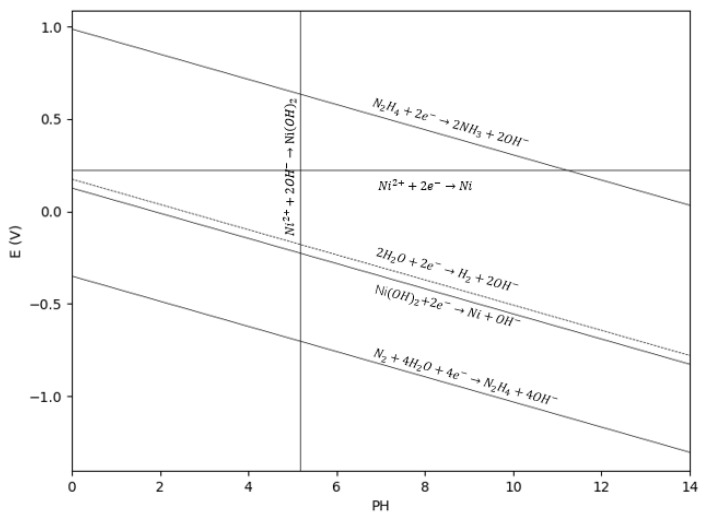
E-pH diagram of the cathodic half-reactions for the liquid-phase reduction of nickel at 70 °C.

After correcting the standard electrode potentials for each reaction at 70 °C using the Gibbs free energy equation, the variation trends of the electrode potentials (*E*) for the aforementioned half-reactions as a function of pH were calculated via the Nernst equation, as illustrated in Figure 1.

### 3.2. Particle Size Regulation of Nano-Nickel

#### 3.2.1. Effect of Alkanolamine Compound Concentration on Nano-Nickel

**Figure 2:**
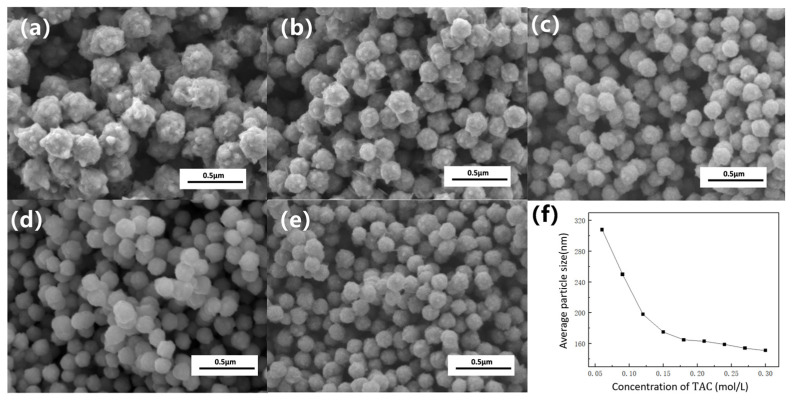
SEM images of nano-nickel powders synthesized with varying concentrations of the alkanolamine complexing agent (TAC): (**a**) 0.06 mol/L; (**b**) 0.12 mol/L; (**c**) 0.15 mol/L; (**d**) 0.18 mol/L; (**e**) 0.24 mol/L; (**f**) average particle size of the nano-nickel powders as a function of TAC concentration.

We systematically investigated the effect of an alkanolamine complexing agent (TAC) on the particle size and morphology of the products under standard conditions (0.85 mol/L hydrazine, pH 14.3). As shown in the SEM images (Figure 2), a low TAC concentration (0.06 mol/L) yields quasi-spherical particles with numerous surface burrs and a large average size of ~268 nm. Increasing the TAC concentration progressively refines both morphology and size. At 0.24 mol/L TAC, the particles become highly spherical, the size decreases to ~132 nm, and the surface burrs are essentially eliminated.

**Figure 3:**
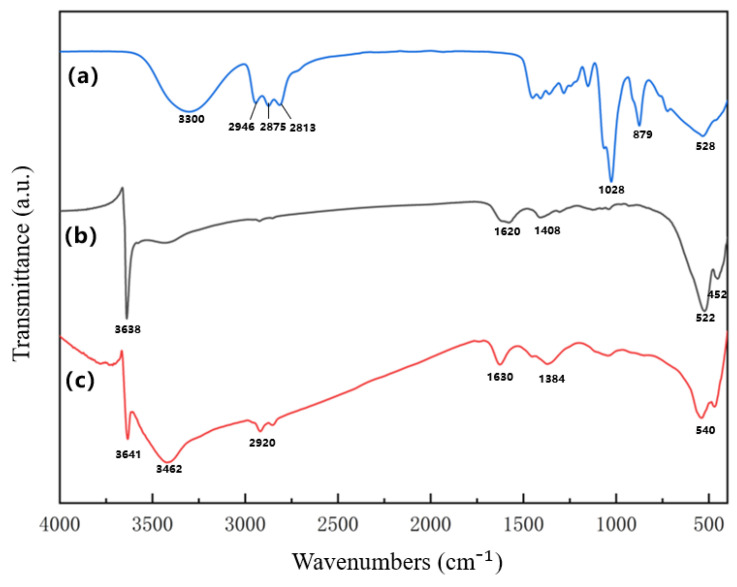
FTIR spectra of the pure alkanolamine complexing agent (TAC) and the synthesized nano-nickel powders: (**a**) pure TAC reagent; (**b**) unmodified nano-nickel; (**c**) nano-nickel synthesized with 0.48 mol/L TAC.

Figure 3 presents the FTIR spectra of the reagents and the synthesized nano-nickel. Without TAC (b), the broad peak at ~3640 cm^−1^ corresponds to isolated surface hydroxyls (O–H), indicating residual Ni(OH)_2_. The ~1620 cm^−1^ peak represents the H–O–H bending of adsorbed water, while the peaks below 600 cm^−1^ denote Ni–O lattice vibrations. For the sample modified with 0.48 mol/L TAC (c), new peaks emerge at 3462 cm^−1^ (O–H stretching) and 2920 cm^−1^ (C–H stretching), confirming the presence of TAC on the particle surface. A minor peak at ~1020 cm^−1^ (C–O stretching) suggests minimal carbon-oxygen bonding. These spectral shifts indicate that TAC avoids direct reduction and instead strongly adsorbs onto the nickel surface via O–H groups, altering the local chemical environment. This strong surface interaction is further corroborated by the disappearance of the ~897 cm^−1^ peak, which corresponds to the skeletal vibration of the TAC molecule. Macroscopically, increasing the TAC concentration visibly intensifies nitrogen gas evolution (Equation (10)), reflecting a higher reaction rate. This kinetic enhancement primarily originates from TAC forming stable multidentate complexes, [Ni${\left(\mathrm{TAC}\right)}_{x}$]^2+^ [25], with nickel ions. In strongly alkaline media, nickel typically precipitates as Ni(OH)_2_, which is reduced much more slowly than soluble nickel species. The [Ni${\left(\mathrm{TAC}\right)}_{x}$]^2+^ complexes temporarily stabilize soluble nickel at high pH, maintaining a rapid initial reduction rate. Furthermore, the adsorption of TAC onto the nano-nickel nuclei suppresses the catalytic self-decomposition of hydrazine, improving the overall reduction efficiency. According to the LaMer model, this accelerated reaction increases supersaturation, triggering a burst nucleation event. Consequently, the heavy consumption of nickel during nucleation depletes the precursor available for the growth stage, restricting particle growth and yielding smaller final particle sizes [26].

**Figure 4:**
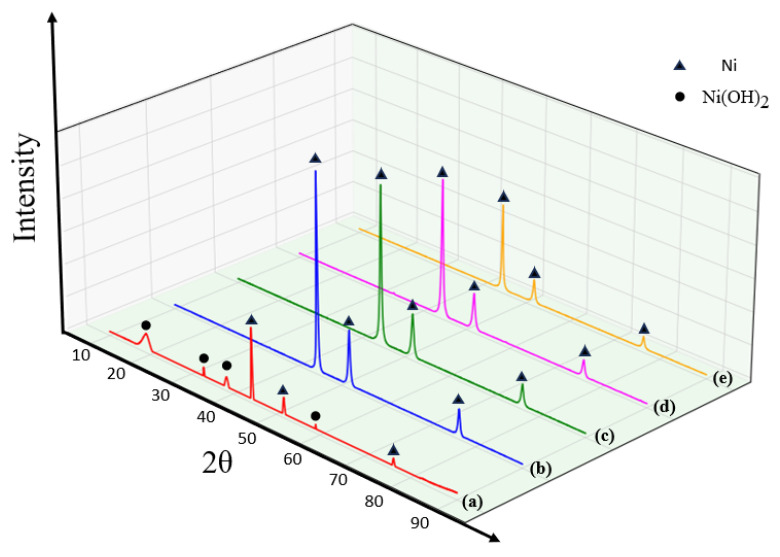
XRD patterns of nano-nickel powders synthesized with varying concentrations of the alkanolamine complexing agent (TAC): (**a**) 0 mol/L; (**b**) 0.06 mol/L; (c) 0.15 mol/L; (**d**) 0.24 mol/L; (**e**) 0.48 mol/L.

Figure 4 displays the XRD patterns of nano-nickel synthesized at varying TAC concentrations. Without TAC (a), the incomplete reduction of Ni(OH)_2_ yields a mixed-phase product. With TAC additions (b–e), only pure FCC Ni peaks are observed. However, as the TAC concentration increases, the diffraction peaks broaden and their intensity decreases, indicating lower crystallinity. According to Ostwald ripening, high-energy surface atoms tend to dissolve and redeposit onto lower-energy grain boundaries to minimize overall surface energy. The encapsulation of the nano-nickel nuclei by TAC sterically hinders this atomic migration and subsequent grain growth, which ultimately accounts for the slight decrease in crystallinity.

#### 3.2.2. Effect of Inorganic Sulfur Salt Concentration on Nano-Nickel

**Figure 5:**
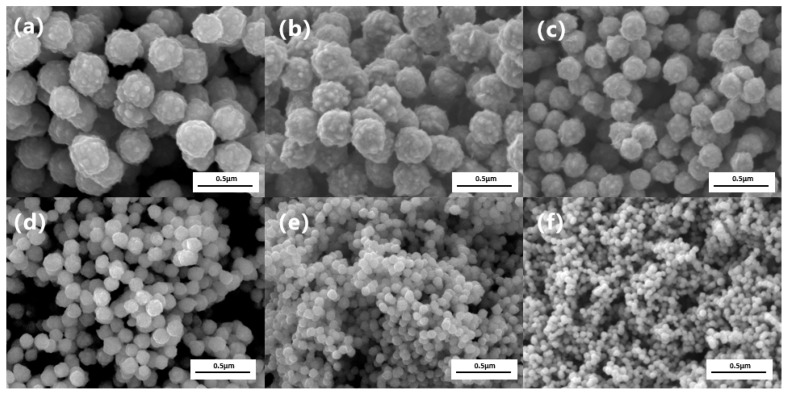
SEM images of nano-nickel powders synthesized with varying concentrations of the inorganic sulfur salt (ISP): (**a**) 0 μL; (**b**) 0.35 μL; (**c**) 1 μL; (**d**) 2.5 μL; (**e**) 12.5 μL; (**f**) 50 μL.

**Figure 6:**
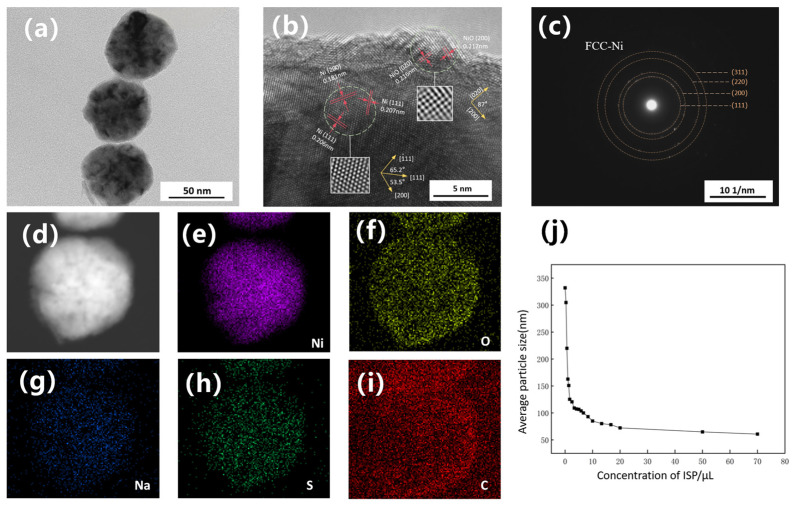
(**a**) Bright-field TEM image of the nano-nickel powder synthesized with 50 μL of ISP; (**b**) corresponding HRTEM image; (**c**) selected area electron diffraction (SAED) pattern of the entire particle; (**d**–**i**) elemental mapping images; and (**j**) average particle size of the nano-nickel powders as a function of ISP concentration.

To understand and effectively control the particle size of nano-nickel synthesized via liquid-phase reduction, this study systematically investigated the influence of the concentration of an inorganic sulfur salt (ISP) on the size and micro-morphology of the final products. Figure 5 presents the SEM images of nano-nickel powders synthesized with varying ISP concentrations under standard reaction conditions (1.3 mol/L hydrazine, pH 14.3), where each microliter of the added ISP solution contains 0.5 $\times \;10^{-8}$ mol of the solute. The SEM characterization reveals that introducing trace amounts of ISP significantly refines both the particle size and surface morphology. The unmodified control group (a) exhibits relatively large (~332 nm) spherical particles with rough, hill-like surface protrusions. Upon the addition of trace ISP (e.g., 1 μL in Figure 5c and Figure 2.5 μL in Figure 5d), the particle size decreases drastically while maintaining a quasi-spherical shape, and the surface protrusions are visibly smoothed. At an ISP dosage of 50 μL, the average particle size of the nano-nickel drops to 64.5 nm. The average particle sizes derived from the SEM images across different ISP concentrations are plotted in Figure 6j. In the low-concentration regime (~0–10 µL), the particle size drops precipitously as the concentration increases. In the intermediate-concentration regime (~10–20 µL), the rate of size reduction notably decelerates. Subsequently, in the high-concentration regime (>20 µL), the size reduction plateaus, undergoing only marginal changes before ultimately stabilizing at approximately 60 nm. These results indicate that adjusting the ISP concentration enables effective control of the nano-nickel particle size within the 300–60 nm range. Although this approach provides higher precision than TAC, the two additives are not mutually exclusive. TAC primarily accelerates reaction kinetics and increases supersaturation to trigger burst nucleation. This enables coarse size tuning at the hundred-nanometer scale and ensures a complete reaction. When targeted particle sizes fall below 100 nm and the sensitivity of TAC decreases, ISP can be introduced for finer adjustment.

Figure 6a displays the nano-nickel powder modified with 50 μL of ISP, and Figure 6c exhibits the corresponding selected area electron diffraction (SAED) pattern for the entire particle. The pattern shows clear, discontinuous concentric diffraction rings, which are characteristic of a typical polycrystalline structure. By calculating the interplanar spacings and radius ratios, these diffraction rings correspond well—from inside to outside—to the (111), (200), (220), and (311) crystal planes of face-centered cubic (FCC) nickel, with no impurity rings observed. Figure 6b presents an HRTEM image of the edge region of a single nanoparticle. The inner core region displays well-resolved lattice fringes, indicating high crystallinity. The measured interplanar spacings are 0.207 nm and 0.181 nm, corresponding to the (111) and (200) planes of FCC metallic nickel, respectively. The characteristic 53.5° interfacial angle between the (111) and (200) planes further confirms the phase structure of the metallic core. In the outer layer, the spacings of 0.216 nm and 0.217 nm are assigned to the (020) and (200) planes of cubic NiO. The nearly perpendicular angle between these planes is consistent with the cubic symmetry of the NiO rock-salt structure, which naturally arises from the spontaneous passivation of the sample in air.

### 3.3. Regulation of Dispersion and Morphology of Nano-Nickel

#### 3.3.1. Multidentate Carboxylate (NNA) System

**Figure 7:**
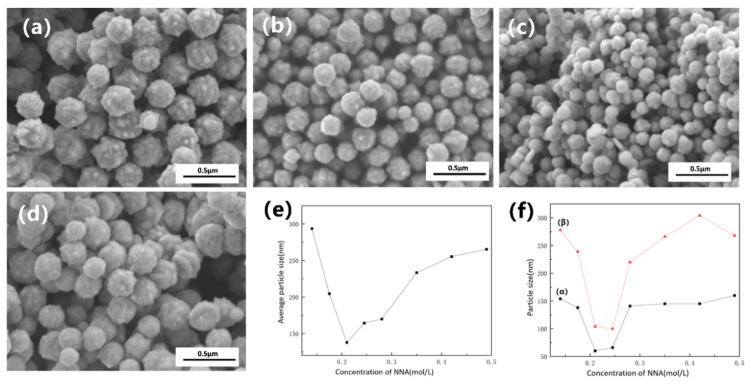
(**a**–**d**) SEM images of nano-nickel powders dispersed with varying concentrations of a multidentate carboxylate (NNA): (**a**) 0.14 mol/L; (**b**) 0.175 mol/L; (**c**) 0.21 mol/L; and (**d**) 0.49 mol/L; (**e**) average particle size of the nano-nickel powders as a function of NNA concentration; and (**f**) effect of NNA concentration on the dispersion performance (α: D10; β: D50).

In a rapid, hydrazine-driven reduction system, kinetic control alone is insufficient to completely prevent the high-energy agglomeration of nanoparticles. Therefore, this section systematically investigates the effect of varying concentrations of a multidentate carboxylate (NNA) on the dispersion properties and micro-morphology of the final products. Figure 7a–d presents SEM images of nano-nickel powders dispersed with different NNA concentrations. Figure 7e,f illustrates the impact of NNA concentration on the particle size and dispersion performance, respectively, where (α) and (β) represent the D10 and D50 dispersion reference indices. As the NNA concentration increases from 0.14 mol/L to 0.49 mol/L, the nickel particle size exhibits a U-shaped trend—initially decreasing and subsequently increasing—reaching a minimum of ~140 nm at 0.21 mol/L. At this optimal concentration, the particle dispersibility is also maximized (i.e., yielding the lowest D50 value, as shown in Figure 7f). This phenomenon reflects a shift in the dominant regulatory mechanism of NNA across different concentration regimes.

During the liquid-phase synthesis of nano-nickel, NNA acts simultaneously as both a complexing agent and a dispersant. As a multidentate carboxylate ligand, the NNA anion can coordinate with Ni^2+^ to form thermodynamically stable, water-soluble nickel complexes (primarily in forms such as [Ni(NNA)]^−^ or [Ni(HNNA)(H_2_O)_n_]). In a strongly alkaline environment, hydroxide ions compete for the coordinated Ni^2+^. However, due to the inherently rapid reaction kinetics, the NNA complex temporarily sustains a high concentration of soluble Ni^2+^ during the initial reaction stage. In a high-pH system lacking effective complexing agents, Ni^2+^ preferentially converts into solid-phase nickel hydroxide. This forces the reaction into a kinetically sluggish and inefficient solid–liquid reduction pathway, where the reaction rate is strictly limited by the solid surface area and slow mass transfer processes. The introduction of NNA transiently captures Ni^2+^ before hydroxide precipitation can occur, ensuring highly efficient and rapid contact between hydrazine and soluble Ni^2+^. Macroscopically, this leads to an accelerated overall reaction rate, elevated supersaturation, and consequently, a smaller final particle size. Following the nucleation and initial growth of nickel atoms into primary nanoparticles, the NNA anions rapidly adsorb onto the nascent metal surfaces, transitioning into the role of a dispersant. This adsorption, primarily mediated by multiple deprotonated carboxyl groups (–COO^−^), constructs a dense electrical double layer on the nanoparticle surface. This significantly elevates the surface Zeta potential, effectively counteracting the van der Waals attractive forces that drive agglomeration, thereby enhancing the dispersibility of the nano-nickel powder. According to classical DLVO theory for colloidal stability, the height of the electrostatic repulsion barrier and the effective thickness of the electrical double layer are inversely proportional to the ionic concentration of the solution. When the NNA concentration is excessively high, the increased ionic strength severely compresses the electrical double layer on the nanoparticles, shortening the range of electrostatic repulsion. Similarly, Afshinnia et al. [27] reported that an excess concentration of carboxylates during the dispersion of silver nanoparticles via charge effects leads to a reduction in double-layer thickness. Once the repulsive barrier drops below the threshold required to overcome van der Waals attractions, the system exceeds the critical coagulation concentration. At this point, the previously dispersed nanoparticles begin to re-agglomerate, which is macroscopically reflected by a corresponding increase in the D50 and D10 values.

Although introducing an appropriate amount of multidentate carboxylate (NNA) effectively optimizes the dispersion and particle size of the nano-nickel, the complexation and electrostatic repulsion provided by the NNA anions cannot intrinsically suppress the anisotropic growth of surface burrs. The apparent reduction in burrs with increasing NNA concentration, as observed in Figure 7a–d, is actually a secondary effect driven by the accelerated reaction rate. A higher instantaneous supersaturation triggers burst nucleation, generating a massive number of initial nuclei. Consequently, the depleted concentration of residual nickel atoms in the solution becomes insufficient to sustain long-distance diffusion and anisotropic growth, which macroscopically results in fewer burrs. Therefore, the fundamental issue of surface morphology remains unresolved.

**Figure 8:**
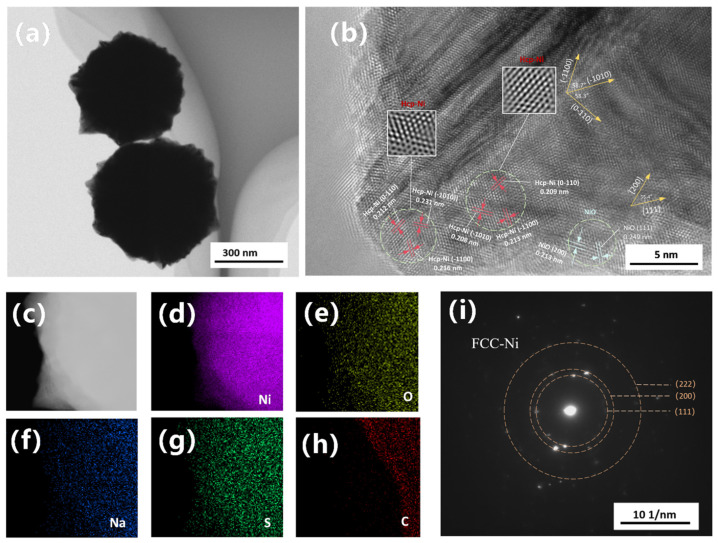
(**a**) Bright-field TEM image of the spiky nano-nickel powder; (**b**) HRTEM image of the burr region; (**c**–**h**) elemental mapping images of the nano-nickel; and (**i**) selected area electron diffraction (SAED) pattern of the nano-nickel.

The formation of such unique urchin-like or spiky nickel nanostructures typically involves complex interfacial growth kinetics. Eluri et al. [28] suggested that under strictly reducing atmospheres (e.g., high-pressure hydrogen or pure nitrogen), the initial spiky structures result from the deposition and anisotropic growth of metallic nickel on the surface matrix, which subsequently forms a 1–5 nm NiO passivation layer upon exposure to air. Meanwhile, Peck et al. [29] pointed out that analyzing nano-NiO solely using XPS and XRD is susceptible to interference from surface hydroxyl hydrolysis. The crystallographic origins and formation mechanisms of this anomalous morphology were further characterized using TEM and HRTEM. Figure 8a shows nano-nickel synthesized at a low NNA concentration with an extended reaction time to allow sufficient diffusion of residual nickel atoms, ultimately yielding a distinct core–shell and burr-like structure. The corresponding selected area electron diffraction (SAED) pattern of the entire particle (Figure 8i) reveals two intense innermost diffraction rings. Based on calculations of interplanar spacings and radius ratios, these rings correspond well to the (111) and (200) planes of face-centered cubic (FCC) nickel, confirming that the overall particle maintains a well-defined polycrystalline FCC structure. Figure 8b presents the HRTEM image of the burr region, from which three distinct zones were analyzed: the inner core of the burr, the sub-surface layer, and the surface oxide layer. To avoid excessively low electron beam transmittance deep within the core, a region approximately 10 nm inwards from the burr surface was selected. This inner region exhibits relatively ordered lattice arrangements, albeit with noticeable fringe blurring and contrast fluctuations. Such features typically indicate local strain fields or stacking faults, generated as atoms deviate from their equilibrium positions to accommodate lattice distortion. The measured interplanar spacings for lattices in three distinct directions are 0.208 nm, 0.209 nm, and 0.213 nm. Compared to the standard $\left(10\bar{1}0\right)$ plane of HCP-Ni (d ≈ 0.215 nm [30]), these values exhibit a 2–3% contraction, which is likely attributable to nanoscale lattice distortion or compressive stress. Crucially, the interfacial angles between these three planes are 58.7° and 58.3°. In contrast to the theoretical 70.5° angle of FCC-Ni {111} planes and the 54.7° angle between NiO {200} and {111} planes, these measured angles align most closely with the theoretical 60° of an HCP structure (error ≤ 3%), exhibiting HCP-like atomic arrangement features when viewed along the [0001] zone axis, with the three crystal planes corresponding to $\left(\bar{1}010\right)$, $\left(0\bar{1}10\right)$, and $\left(\bar{1}100\right)$. Furthermore, the variation in interplanar spacings along different directions within the same grain confirms the presence of a non-uniform stress field within the burrs. The region closer to the burr tip maintains the HCP skeleton. However, owing to its proximity to the NiO layer—which possesses a larger crystal lattice—its interplanar spacings, particularly along the $\left(10\bar{1}0\right)$ direction, are subjected to tensile strain. The outermost surface of the burr consists of a 1–3 nm thick NiO passivation layer. This is well-confirmed by interplanar spacings of 0.213 nm and a characteristic 0.249 nm, corresponding to the NiO (200) and (111) planes, respectively, with an interfacial angle of 55.4°. The distinct outlines of oxygen and other elements within the burrs, as shown in the elemental mapping (Figure 8c–h), further corroborate this observation. Given the extremely high surface energy of nano-nickel, this Ni/NiO structure is virtually inevitable. The NiO layer on the particle surface is formed via rapid oxidation when the nano-nickel powder is exposed to room-temperature air after vacuum drying. It is typically not a perfectly encapsulating single-crystal shell, but rather a polycrystalline structure assembled from multiple randomly oriented nanocrystallites. Shi et al. [31] pointed out that in MLCC production, this ultra-thin oxide layer helps improve the oxidation resistance of the particles, while simultaneously enhancing thermal shrinkage resistance and suppressing abnormal particle sintering.

**Figure 9:**
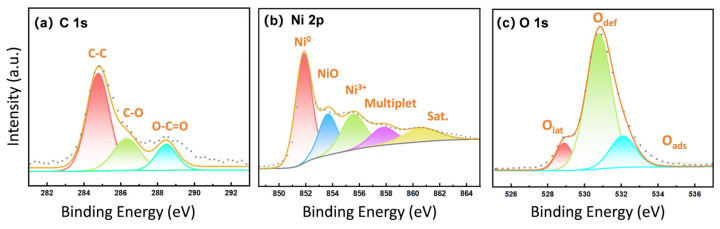
XPS spectrum of the spiky nano-nickel powder (**a**) C 1s; (**b**) Ni 2p; (**c**) O 1s.

To confirm the formation of metallic nickel and further verify the coexistence of FCC and metastable HCP structures, XPS and ICP analyses were performed on the spiky nano-nickel powder. As shown in Figure 9b, the main peak at 852.6 eV in the high-resolution Ni 2p spectrum is assigned to metallic Ni^0^. The adjacent peak at 853.7 eV (NiO) represents Ni^2+^ in the standard rock-salt structured NiO, while the peak at 855.5 eV (Ni^3+^) points to a defective oxidation state or surface-adsorbed nickel, indicating a polycrystalline NiO shell with abundant defects. The peak at 861.0 eV is the characteristic satellite peak (Sat.) of NiO. The O 1s spectrum (Figure 9c) can be clearly deconvoluted into lattice oxygen (O_lat_), defective oxygen/oxygen vacancies (O_def_), and chemisorbed oxygen (O_ads_). In the C 1s spectrum (Figure 9a), a distinct characteristic peak of the carboxyl group (O-C=O) appears at 288.6 eV, confirming the chemisorption of NNA molecules on the surface of the nanoparticles. Subsequent ICP analysis reveals that the overall purity of the nickel powder reaches up to 99.97 wt.%, with impurity elements such as Na, Fe, Ca, and S all below the detection limit (ND). Combined with the SAED pattern (Figure 8i), this confirms that the bulk of the sample is high-purity FCC-Ni.

The energy difference between FCC and HCP structures fundamentally arises from the stacking fault energy. Under the high supersaturation conditions typical of liquid-phase synthesis, rapid crystallization kinetics can prevent atoms from relaxing into the lowest-energy FCC positions in time. This kinetic limitation generates abundant stacking faults. When the local density of these stacking faults becomes sufficiently high, the long-range ABCABC stacking sequence is disrupted and locally replaced by an ABAB sequence, manifesting macroscopically as localized HCP structures. Localized HCP nickel nanocrystals typically form via an amorphous-phase-mediated mechanism [32]. Driven by high reactivity, the solution undergoes spinodal decomposition into Ni-rich and Ni-poor liquid phases, creating a highly concentrated local environment that provides the thermodynamic and kinetic basis for stacking fault generation. Liu et al. [33] calculated that the surface energy of the low-index Ni (0001) facet of the HCP type is lower than that of the FCC-Ni (111) facet. Density functional theory (DFT) calculations by Zhang et al. [32] demonstrated that the adsorption energies of the HCP-Ni $\left(\bar{1}010\right)$ and (0001) facets are significantly lower than those of other crystal planes (FCC-Ni (100), (110), (111), and HCP-Ni $\left(11\bar{2}0\right)$. From a thermodynamic perspective, molecules and atoms within the reaction system exhibit a strong tendency for selective crystallization on the HCP-Ni $\left(\bar{1}010\right)$ and (0001) facets. This is highly consistent with our previous discussion: in the absence of a strong steric hindrance agent, the high thermodynamic reactivity of the $\left(\bar{1}010\right)$ facet drives rapid unidirectional extension, ultimately growing into a spiky structure macroscopically. Furthermore, Zhang experimentally observed that crystal nucleation predominantly occurs at the periphery of these Ni-rich liquid domains, where nickel atoms preferentially attach to the HCP-Ni $\left(\bar{1}010\right)$ facets for layer-by-layer growth. This specific facet-directed growth mode leads to the rapid extension of the crystal in a single direction, which perfectly explains the distinct elongation of lattice fringes observed in the high-resolution images of the burr regions.” Ding et al. [34] demonstrated that platinum (Pt)—which shares similar slip systems and stacking fault energy characteristics with Ni—forms Shockley partial dislocations at its surface under stress. As these partial dislocations glide across the crystal planes, they leave behind stacking faults and deformation twins within the crystal interior. A similar stress-induced dislocation mechanism is highly likely to be a contributing factor to the morphological evolution of the spiky surface in these nano-nickel particles.

#### 3.3.2. Thioether-Based Amino Acid (TAA) System

**Figure 10:**
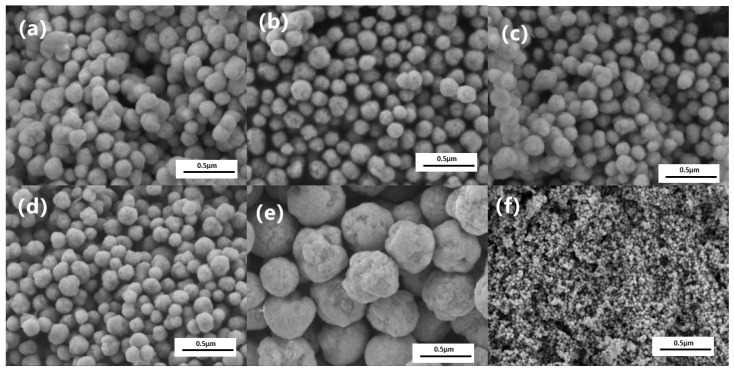
(**a**–**d**) SEM images of nano-nickel powders dispersed with varying concentrations of a thioether-based amino acid (TAA): (**a**) 0.14 mol/L; (**b**) 0.20 mol/L; (**c**) 0.24 mol/L; and (**d**) 0.337 mol/L; (**e**,**f**) SEM images of nano-nickel powders synthesized with inorganic sulfur salt (ISP) additions of (**e**) 0 μL and (**f**) 200 μL.

**Figure 11:**
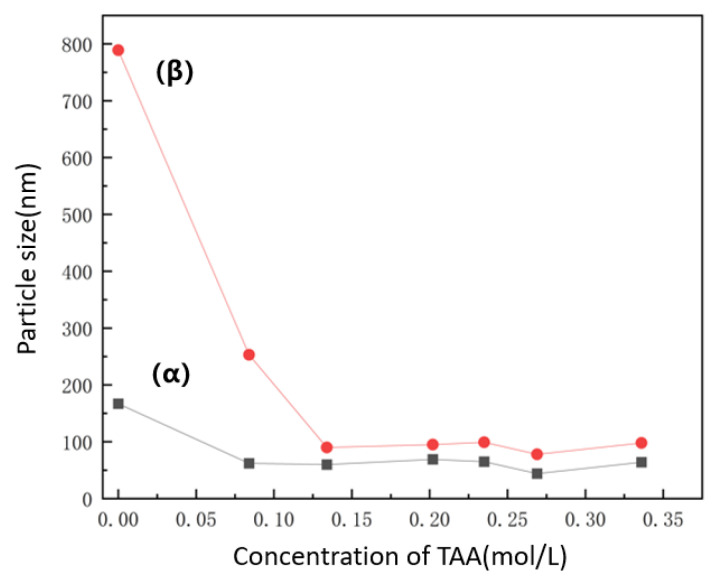
Effect of TAA concentration on the dispersion performance of the nano-nickel powders (α: D10; β: D50).

To address the issue of surface burrs within the system, this section systematically investigates the application of a thioether-based amino acid (TAA). Figure 10a–d displays the SEM images of nano-nickel powders dispersed with varying TAA concentrations. The particles exhibit an overall spherical morphology. Unlike the multidentate carboxylate (NNA) dispersed samples, the nano-nickel synthesized with TAA features a burr-free surface with only minor pitting. TAA effectively disperses the nano-nickel powders, resulting in no obvious interparticle adhesion. Variations in TAA concentration have a negligible impact on particle size. Because the steric hindrance effect of TAA is significantly stronger than the electrostatic repulsion of NNA, its dispersion performance is correspondingly superior. Figure 11 illustrates the effect of TAA concentration on dispersion performance; optimal dispersion is achieved at concentrations above 0.24 mol/L, and excessive TAA does not induce additional agglomeration. Notably, within this dispersion system, the particle size can still be precisely controlled via the inorganic sulfur salt (ISP). Figure 10e,f presents SEM images of nano-nickel synthesized with varying ISP concentrations under TAA dispersion. This coupled system enables the precise preparation of nickel powders ranging from 50 nm to 600 nm, maintaining a reaction yield exceeding 90%.

The TAA molecule offers three potential coordination sites for the borderline acid Ni^2+^: a hard amino nitrogen atom, a hard carboxyl oxygen atom, and a soft thioether sulfur atom. Hard and Soft Acids and Bases (HSAB) theory dictates that strong interactions predominantly occur between hard acids and hard bases, or between soft acids and soft bases. Experimental data, such as NMR studies by Berthon et al. [35], confirm that the interaction between the sulfur atom (a soft Lewis base) and Ni^2+^ (a borderline Lewis acid) is exceedingly weak; thus, the thioether group essentially does not participate in Ni^2+^ coordination. For Ni^2+^, TAA primarily acts as a bidentate ligand governed by the chelate effect. It coordinates with the nickel ion via its amino nitrogen and carboxyl oxygen atoms, forming a thermodynamically highly stable five-membered chelate ring. The formation of this ring is entropically highly favorable, as one bidentate ligand replaces two monodentate water molecules, thereby increasing the total number of particles in the system and driving up the entropy. This specific coordination configuration also explains the experimental observation that prematurely mixing TAA with the nickel precursor severely impedes the reduction reaction.

TAA effectively disperses the nanoparticles through both steric hindrance and electrostatic repulsion. Once nucleation commences and Ni^2+^ is reduced to Ni^0^, the uncoordinated sulfur in the thioether side chain serves as the primary adsorption site on the surface of Ni^0^ (a soft acid). Together with the nitrogen and oxygen atoms, it forms a robust multi-point surface bond with the nickel. Nemkovade et al. [36] pointed out that the binding between such amino acids and metal surfaces is exceptionally strong. Furthermore, Yeganeh et al. [37] confirmed that these amino acids can spontaneously form a dense molecular coating layer on the surface of the nanoparticles. This layer provides high stability to the system and physically restricts the epitaxial growth of the particles. Density functional theory (DFT) and molecular dynamics (MD) simulations by Gupta et al. [38] revealed that the adsorption energy of such amino acids on the Ni (111) surface is approximately −1.50 eV. The molecule adopts a highly stable flat-lying adsorption configuration on the Ni (111) facet, accompanied by charge transfer that leaves the nickel surface positively charged. Additionally, its long side chain (-CH_2_-CH_2_-S-CH_3_) physically screens adjacent active sites, hindering further Ni atom deposition and dispersing the nanoparticles via steric hindrance [39], thereby significantly suppressing the formation of surface burrs.

**Figure 12:**
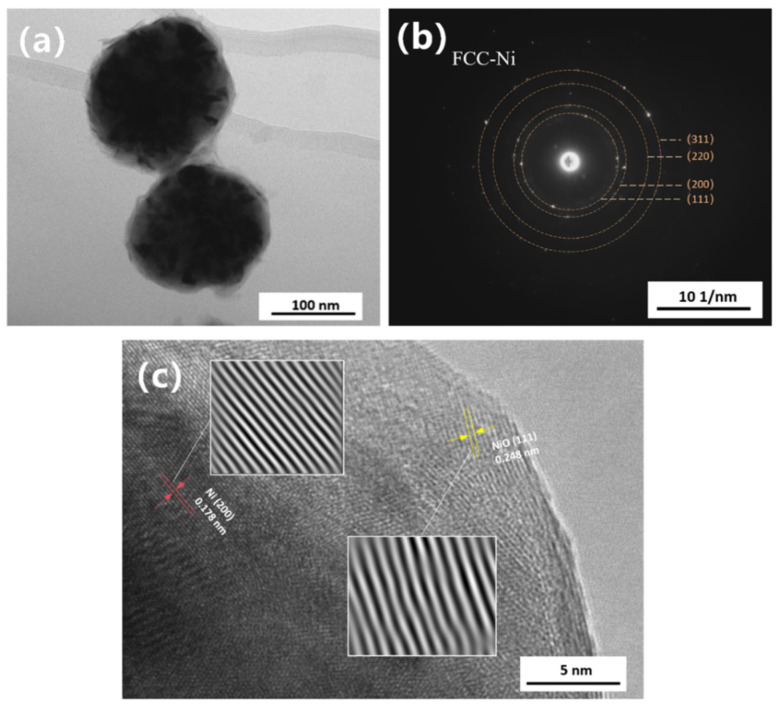
(**a**) Bright-field TEM image of the nano-nickel powder synthesized with TAA; (**b**) selected area electron diffraction (SAED) pattern of the entire particle; and (**c**) corresponding HRTEM image.

Figure 12a presents a bright-field TEM image of the nano-nickel powder synthesized with TAA assistance. The particles exhibit a quasi-spherical morphology with slightly rough edges, completely devoid of obvious dendritic or burr structures. Minor step-like fluctuations on the surface are attributed to competitive growth caused by differing crystal plane growth rates. The uneven internal contrast indicates that the particle is assembled from numerous primary crystallites with varying orientations. Figure 12b shows the selected area electron diffraction (SAED) pattern. The radii and ratios of the concentric rings, which are composed of discrete diffraction spots, highly align with the theoretical data for FCC-Ni. The particle as a whole is a polycrystalline aggregate with a Ni/NiO core–shell structure, dominated by aggregative growth. The HRTEM image in Figure 12c clearly unveils the microstructural details of the particles. It distinctly resolves the characteristic (200) crystal planes within the metallic Ni core and the NiO (111) planes in the surface oxide layer. The measured interplanar spacings deviate from standard values by approximately 2%; this is likely caused by minor lattice distortions induced by the nanoscale size effect or surface defects. This distortion is particularly pronounced in the near-surface NiO layer, where distinct bending of the lattice fringes is observed.

### 3.4. Nucleation and Growth of Nano-Nickel

The nucleation and growth of nano-nickel follow the classical LaMer model. Under the action of a strong reducing agent, Ni^2+^ in the solution is continuously reduced to Ni^0^. As the Ni^0^ monomers continuously generate and accumulate, the system surpasses the critical supersaturation and overcomes the nucleation energy barrier, thereby triggering burst nucleation. Subsequently, the reaction transitions from a homogeneous nucleation-dominated regime to a diffusion-controlled heterogeneous growth and spontaneous agglomeration stage. However, the steady progression of this macroscopic kinetic model relies on a stable supply of free Ni^2+^ within the system, which is exceptionally scarce in an actual high-pH reaction environment.

**Figure 13:**
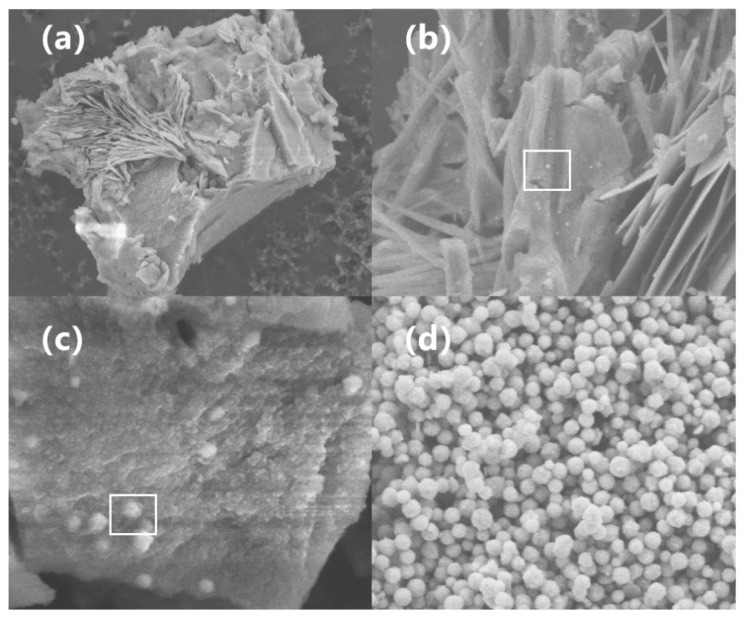
SEM images of nano-nickel powders synthesized at different reaction times: (**a**) 1 min; (**b**) 4 min; (**c**) 7 min; and (**d**) 25 min after the initiation of the reaction.

As indicated by the E-pH diagram in Figure 1, when the system pH exceeds 5.3, Ni^2+^ preferentially combines with hydroxide ions in the solution to form a matcha-green Ni(OH)_2_ precipitate. This precipitate is extremely stable, with a standard K_SP_ of 5.48 × 10^−16^. At pH 14.2, the concentration of free Ni^2+^ in the solution plummets to approximately 2.18 × 10^−16^, which is negligible for direct reduction. Consequently, the nickel species predominantly exist in the form of Ni(OH)_2_. Because the thermodynamic driving force for the reduction of free Ni^2+^ is vastly greater than that for Ni(OH)_2_, the reducing agent will only target Ni(OH)_2_ when free Ni^2+^ is depleted, making the soluble Ni^2+^ concentration the primary bottleneck limiting the overall reaction rate. Table 1 compares the required nucleation times for two distinct coordination scenarios: (1) thoroughly mixing hydrazine hydrate with nickel sulfate to form a complex (denoted as [Ni(N_2_H_4_)_n_]^2+^ prior to adding sodium hydroxide to initiate the reaction, versus (2) thoroughly mixing sodium hydroxide with nickel sulfate to form Ni(OH)_2_ prior to adding hydrazine hydrate. Although all other parameters for these two addition sequences were identical, the time required for the solution to turn black (indicating the onset of nucleation) differed significantly. This implies that the actual function of the complex is to kinetically compete with hydroxide ions; owing to its moderate coordination strength, it can temporarily shield a fraction of the Ni^2+^ ions in a strongly alkaline environment. Each coordinated Ni^2+^ serves as a potential reaction site with a lower transition state barrier for intramolecular electron transfer, rendering the coordinated Ni^2+^ much easier to reduce. This fundamentally explains the significant discrepancy in reaction rates between different complex types under the identical hydrazine hydrate concentrations. The overall nucleation and growth process is illustrated in Figure 13: the Ni^2+^ ions—either dissolving from the layered Ni(OH)_2_ or slowly released from the complexes—are preferentially reduced to form primary nuclei, which subsequently grow within the Ni(OH)_2_ layers [17,40] until the Ni(OH)_2_ precursor is completely consumed.

## 4. Conclusions

In this study, we investigated the regulatory mechanisms of functional additives on the nucleation and growth kinetics, as well as the microstructural evolution, of nano-nickel powders synthesized via hydrazine-driven liquid-phase reduction.

The results demonstrate that an alkanolamine complexing agent (TAC) effectively modulates the reduction rate in the liquid-phase system, leading to a significant decrease in the average particle size. Compared to the kinetically sluggish solid–liquid reduction pathway, the [Ni${\left(\mathrm{TAC}\right)}_{x}$]^2+^ complexes formed between TAC and Ni^2+^ effectively inhibit the precipitation of nickel hydroxide; this markedly accelerates the reduction kinetics and enhances particle sphericity.

By tuning the concentration of an inorganic sulfur salt (ISP), the particle size of the nano-nickel powders can be precisely and continuously controlled within the 60 nm to 300 nm range, ultimately stabilizing at approximately 60 nm at high concentrations.

Furthermore, highly dispersed spiky spherical nickel powders were obtained by leveraging the electrical double-layer electrostatic repulsion of a multidentate carboxylate (NNA). HRTEM observations revealed that the local atomic arrangement within these surface burrs exhibits metastable HCP characteristics, which is driven by high-density stacking faults.

In contrast, the thioether-based amino acid (TAA) achieves superior dispersion stability and suppresses the anisotropic growth of surface burrs. This is attributed to the strong steric hindrance of its long side chains and selective adsorption onto high-energy crystal facets.

This work provides a practical strategy and theoretical basis for the liquid-phase synthesis of high-performance MLCC inner electrode nano-nickel.

## Figures and Tables

**Table 1:** Nucleation time required for different coordination types.

Type	$N_{2}H_{4}:{Ni}^{2+}$ = 1.3	$N_{2}H_{4}:{Ni}^{2+}$ = 2	$N_{2}H_{4}:{Ni}^{2+}$ = 3	$N_{2}H_{4}:{Ni}^{2+}$ = 4
${\left[Ni{\left(N_{2}H_{4}\right)}_{n}\right]}^{2+}$	1 min 30s	1 min 30 s	1 min 15 s	1 min 10 s
${Ni(OH)}_{2}$	No reaction	6 min 50 s	6 min 30 s	5 min 50 s

## Data Availability

The data presented in this study are available on request from the corresponding author. The raw data are not publicly available due to commercial confidentiality restrictions regarding the specific functional additives utilized in this research.
